# Modulation of interleukin-6 and its effect on late vein wall injury in a stasis mouse model of deep vein thrombosis

**DOI:** 10.1016/j.jvssci.2022.04.001

**Published:** 2022-04-22

**Authors:** Abigail R. Dowling, Catherine E. Luke, Qing Cai, Antonio M. Pellerito, Andrea T. Obi, Peter K. Henke

**Affiliations:** a Conrad Jobst Vascular Surgery Research Laboratories, Department of Surgery, College of Medicine, University of Michigan, Ann Arbor, MI; b Vascular Surgery Section, Department of Surgery, College of Medicine, University of Michigan, Ann Arbor, MI

**Keywords:** Deep vein thrombosis, Interleukin-6, Lovenox, Low-molecular-weight heparin, Vein wall remodeling, Venous thrombosis

## Abstract

**Objective:**

Deep vein thrombosis (DVT) and its sequela, post-thrombotic syndrome (PTS), remain a clinically significant problem. Interleukin-6 (IL-6) is a proinflammatory cytokine that is elevated in patients who develop PTS. We hypothesized that genetic deletion of IL-6 and the use of anti–IL-6 pharmacologic agents would be associated with decreased late vein wall injury.

**Methods:**

Wild-type C57BL/6J (WT) and IL-6^−/−^ mice underwent induction of stasis venous thrombosis by ligation of the infrarenal IVC. Vein wall inferior vena cava and thrombus were harvested at 21 days after ligation and analyzed by Western blot and immunohistochemistry of the vein wall using monocyte markers CCR2 and arginase 1, the endothelial marker CD31, and fibroblast markers DDR2 and FSP-1. Two anti–IL-6 pharmacologic agents (gp130 [glycoprotein 130] and tocilizumab) were tested and compared with low-molecular-weight heparin (LMWH) as the reference standard in WT mice. Plasma was collected at 4 and 48 hours to confirm the pharmacologic agents’ effects.

**Results:**

Less fibrosis but no increase in luminal endothelialization was found in IL-6^−/−^ mice compared with WT mice at 21 days. The IL-6^−/−^ mice had fewer DDR2- and arginase 1-positive cells in the vein wall compared with the WT mice. However, no difference was found in the CCR2^+^ cells. Despite documented in vivo activity, exogenous gp130 and tocilizumab were not associated with decreased vein wall fibrosis or increased endothelial luminal coverage at 21 days. LMWH therapy, both before and after treatment, was not associated with decreased vein wall fibrosis at 21 days.

**Conclusions:**

IL-6 genetic deletion was associated with less fibrotic vein wall injury at a late time point, consistent with the PTS timeframe. However, neither the standard of care LMWH nor two available anti–IL-6 agents showed antifibrotic biologic effects in this model.

## Clinical Relevance

Post-thrombotic syndrome (PTS) has no direct therapy. Also, although anticoagulation therapy can prevent recurrent deep vein thrombosis (DVT), it cannot prevent PTS. PTS is a fibrotic disease, and interleukin-6 is a biomarker for incident PTS. We examined, in a stasis mouse model of DVT, the effect of genetically deleting interleukin-6 (IL-6^−/−^) and tested anti–IL-6 agents. Although IL-6^−/−^ was associated with less vein wall injury, the anti–IL-6 agents did not confer a similar effect and were not associated with vein wall healing.

**Article Highlights**

• **Type of Research:** Experimental research using a model of deep vein thrombosis in a mouse to evaluate the global deletion of interleukin-6 (IL-6) and anti–IL-6 agents
• **Key Findings:** In a stasis model of deep vein thrombosis, although global deletion was associated with decreased vein wall fibrosis, anti–IL-6 agents did not affect vein wall fibrosis.
• **Take Home Message:** IL-6 might not play a major role in post-thrombotic syndrome.

Venous thromboembolism, defined as the occurrence of deep vein thrombosis (DVT) or pulmonary embolism, or both, affects an estimated one half million persons in the United States annually, causing considerable morbidity and mortality. Among those who have had DVT, one third to one half will develop long-term post-thrombotic syndrome (PTS), which manifests as chronic pain, swelling, and skin changes in the affected limb.^1^^,^^2^ Anticoagulation therapy is the standard of care, and, although effective, bleeding risks remain, and these agents do not have direct anti-PTS effects.^3^^,^^4^

The intersection between PTS and fibrotic vein wall pathophysiology is complex and involves growth factors, leukocytes, the plasmin axis, and matrix metalloproteinases.^5^ The intersection is only partially understood owing to the limited number of human specimens of post-thrombotic veins.^6^ It is clear from experimental models that the resolving thrombus is an inflammatory nidus, which incorporates into the vein wall as it heals. This creates a thickened and potentially nonfunctional vein wall (eg, less contractability).^7^ The limb symptoms will be worse if the vein segments are fully occluded. However, even if not, valve damage will often result and lead to venous hypertension and the typical signs and symptoms of PTS.^8^

Recent human studies have suggested that circulating IL-6 is a biomarker of incident PTS and DVT burden.^9^^,^^10^ IL-6 signaling is an important part of the healing process, regulating the transition from neutrophil- to monocyte/macrophage-driven inflammatory processes and fibrosis.^11^ Our laboratory, and others, has also shown that IL-6 might play an active role in venous thrombosis (VT) resolution, partly mediated by matrix metalloproteinase activity.^12^^,^^13^ Although impaired thrombus resolution in humans has been correlated with the occurrence of PTS, no effective preventative or treatment strategies are available.^14^^,^^15^ Even the CaVenT (catheter-directed venous thrombolysis in acute iliofemoral vein thrombosis) and ATTRACT (acute venous thrombosis: thrombus removal with adjunctive catheter-directed thrombolysis) active thrombus removal trials have failed to provide evidence to change therapies or that anticoagulation therapy is even effective in preventing PTS.^7^^,^^16^^,^^17^

Given this major gap in direct PTS therapies, we hypothesized that IL-6^−/−^ would have less vein wall fibrotic injury and that exogenous IL-6 inhibition using currently available anti–IL-6 agents would confer a similar biologic effect. Second, we hypothesized the IL-6^−/−^ and exogenous IL-6 inhibition would result in increased endothelial luminal recovery.

## Methods

### Mice

Male mice (C57/BL6J; weight, 20-30 g; wild-type [WT]) were used for all experiments and were purchased from Jackson Laboratories (stock no. 000664; Bar Harbor, ME). The IL-6^−/−^ mice were also purchased from Jackson Laboratories (stock no. 002650; B6.129S2-*Il6*^*tm1Kopf*^/J) and bred in-house. A total of 140 mice were used. The specific mouse numbers were as follows: histologic examination, n = 21; Western blot, n = 13; enzyme-linked immunosorbent assay (ELISA), n = 60; low-molecular-weight heparin (LMWH) histologic examination, n = 24; glycoprotein 140 (gp130), tocilizumab, and control histologic examination, n = 22. For all procedures, the mice underwent general anesthesia with 2.0% to 2.5% isoflurane and oxygen with continuous monitoring on a 37°C heating mat. All animal studies were performed after approval by the University of Michigan institutional animal care and use committee.^18^

### VT mouse model

VT was formed via generation of stasis blood flow by infrarenal inferior vena cava (IVC) ligation.^19^, ^20^, ^21^, ^22^ In brief, the mice were anesthetized via 2% to 2.5% inhaled isoflurane with oxygen gas at 0.5 L/min, and midline laparotomy was performed. The venous side and dorsal branches were interrupted, and the infrarenal IVC was ligated with a 7-0 Prolene suture (Ethicon Inc, Somerville, NJ) to generate stasis thrombosis. The peritoneum was closed with 5-0 Vicryl suture (Ethicon Inc), and the skin incision was secured with skin glue or wound clips (7-mm wound clips; Reflex Inc, San Francisco, CA), and the mice were allowed to recover under a warming lamp. The mice were euthanized on postoperative day 21. Before processing, the IVC and thrombus were measured and weighed en bloc.^18^, ^19^, ^20^

### Sham surgery mice

The control mice were housed and treated under the same conditions as the stasis mice, except that sham surgery was performed. Specifically, the control mice underwent laparotomy with retroperitoneal dissection but without IVC ligation. Similar to the stasis mice, tissue specimens were collected and processed as described for the stasis mice.

### Drug treatment methods

All the studies were performed using C57Bl/6J mice. LMWH (enoxaparin sodium [Lovenox; Sanofi-Aventis, Paris, France]; purchased at the University of Michigan Medical Center Pharmacy) was given at 3 mg/kg subcutaneously for preoperative treatment.^23^^,^^24^ LMWH was given at 6 mg/kg subcutaneously for postoperative treatment.^24^, ^25^, ^26^, ^27^, ^28^ LMWH was given subcutaneously daily starting the day before surgery (before treatment), with the second dose given 1 hour after recovery and continuing until postoperative day 20. Alternatively, the mice were given their first dose at 1 hour after recovery (after treatment) and daily thereafter. The mice were harvested on postoperative day 21.^25^^,^^26^ Tocilizumab (Actemra, Genentech, South San Francisco, CA; purchased from the University of Michigan Medical Center Pharmacy) was given at 0.3 mg/mouse dose intraperitoneally the day before surgery or the day after surgery and then weekly (postoperative days 8 and 15).^29^, ^30^, ^31^, ^32^, ^33^, ^34^, ^35^ Soluble gp130 (recombinant mouse gp130 Fc chimera protein; catalog no. 468-mg-100; R&D Systems, Inc, Minneapolis, MN) was given by intraperitoneal injection at the dose of 2 μg/mouse daily, as described.^36^, ^37^, ^38^, ^39^, ^40^

### Histologic examinations

Fresh tissue (IVC with the thrombus intact) was fixed in 10% neutral buffered formaldehyde for 2 hours, transferred to 70% ethanol, and subsequently embedded in paraffin for immunohistochemistry (5-μm tissue sections; three sections per slide). Antigen retrieval was performed using heat-mediated sodium citrate buffer (10 mM NaCl solution [pH 6.0] at 95°C for 10 minutes and then allowed to cool for 20 minutes). A species-specific polymer reagent kit was used for animal serum nonspecific antigen binding site block and secondary application (Impress Polymer Detection Kits; Vector Laboratories, Burlingame, CA). The tissue sections were stained for anti-DDR2 (1:750; ab76967; Abcam, Cambridge, MA), anti-CCR2 (1:200; Novus Biologicals, Centennial, CO), anti–FSP-1 (1:1500; 07-2274; Millipore Sigma, Burlington, MA), anti-CD31 (1:200; ab28364; Abcam), and anti–arginase-1 (Arg-1; 1:1500; NBP1-54621; Novus Biological, Littleton, CO). A polymer secondary reagent was applied, followed by application of diaminobenzidine equal volume peroxidase substrate (Vector Laboratories, Burlingame, CA). The slides were counterstained with hematoxylin and cover slipped with Cytoseal 60 (Richard-Allan Scientific, Thermo Fisher Scientific, Waltham, MA). The cells were quantified in a blinded fashion, with the positive cells counted using a Nikon B400 bright-field microscope (Nikon, Tokyo, Japan) with a Spot camera (Spot AI, Burlingame, CA) in eight high-power fields (magnification ×1000) radially around the IVC wall, counted, and totaled.^18^

Masson’s trichrome staining for vein wall thickness measurements was performed.^41^ Images were taken using a Nikon B400 bright-field microscope at 100× and analyzed blindly with ImageJ (National Institutes of Health, Bethesda, MD) software. Eight measurements of the blue collagen ring of the vein wall were taken radially around the IVC and averaged, avoiding measurements between the IVC and aorta. The thrombus was not included if it had separated from the vein wall. Three separate sections were analyzed per mouse and averaged. The analyses were performed in a blinded fashion.

### CD31 stained for percentage of reendothelialization

To determine the percentage of reendothelialization with vein wall remodeling, contiguous CD31-stained luminal lengths were measured and analyzed using ImageJ (National Institutes of Health).^42^

### Western blotting

The protein levels of vascular endothelial growth factor 1 (VEGF1) and vascular endothelial cadherin (VECAD) and β-actin were measured by immunoblotting from the vein wall and/or thrombus harvested tissue.^43^ RIPA (radioimmunoprecipitation assay buffer) buffer (Thermo Fisher Scientific, Rockford, IL) and complete ULTRA Mini Tablets (Roche, Mannheim, Germany) to isolate protein from the thrombus and IVC segments. The protein concentration of these lysates was determined using the BCA assay (Thermo Fisher Scientific). Protein separation was achieved by electrophoresis using NuPAGE 10% Bis Tris gels (Invitrogen, Waltham, MA). The proteins were then transferred onto PVDF (polyvinylidene fluoride) membranes (Millipore, Billerica, MA) and probed with the indicated primary antibodies: anti-VECAD (1:500; ab33168; Abcam) and anti-VEGF1 (1:1000; ab32152; Abcam). Bound antibodies were subsequently probed with the indicated secondary antibodies: goat anti-rabbit (1:1000; 7074S; Cell Signaling Technology, Danvers, MA) and goat anti-mouse (1:1000; sc-516102; Santa Cruz Biotechnology, Dallas, TX). Immunoreactive bands were detected using either SuperSignal West Pico Chemiluminescent Substrate (Thermo Fisher Scientific) or a GE Amersham 600 fluorescent imager (GE Healthcare Life Sciences, Piscataway, NJ). Optical densities were normalized to β-actin (1:20,000; sc-47778; Santa Cruz Biotechnology) on PVDF membranes, and densitometry was performed using Image Lab software (Bio-Rad Laboratories, Hercules, CA).

### Plasma ELISA

Whole blood was collected terminally with a cardiac puncture under anesthesia. The needle was removed, and the blood was placed in an appropriately sized lithium heparin or EDTA (ethylenediaminetetraacetic acid) microtainer blood collection tubes (catalog nos. NC9976871 and 5-465-340; Sarstedt Inc, Nümbrecht, Germany), inverted twice, and rocked for <45 minutes. The blood was spun at 2000*g* for 20 minutes at 4°C. The upper plasma was placed into aliquots and stored in a freezer at −20°C for <6 months before performing ELISAs in accordance with the manufacturer’s instructions. The kits used were all Quantikine ELISA immunoassays (R&D Systems, Inc) and included mouse IL-6 and mouse pentraxin 2/serum amyloid P component.

### Statistical analysis

All data are presented as the mean ± standard deviation. Outliers were identified, normality was tested, and normal data were evaluated using the unpaired Student two-tailed *t* test with the Welch correction. One-way analysis of variance with Bonferroni post hoc comparisons were used for comparison between the experimental groups and controls, as appropriate (Prism; GraphPad, San Diego, CA). *P* values of <.05 was considered statistically significant.^18^ The Mann-Whitney *U* test was used to analyze non-normal groups of data.^44^

## Results

### Genetic deletion of IL-6 results in less fibrosis and increased remodeling at a chronic time point

**Fig 1:**
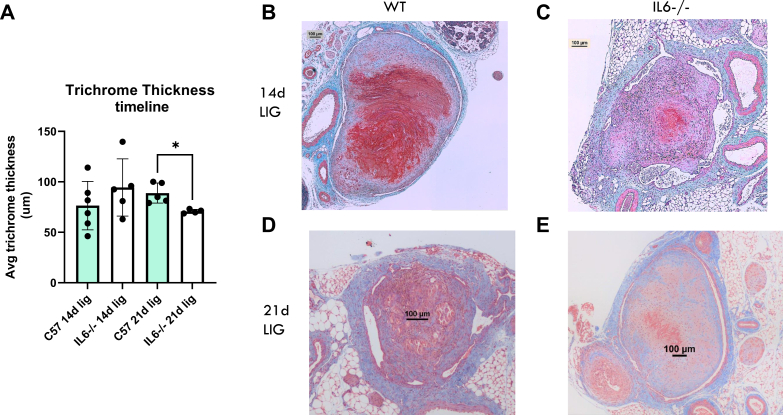
Masson trichrome thickness. **A,** Average trichrome thickness of wild-type (WT) and interleukin-6 deficient (IL-6^−/−^) mice at 14 (*14d*) and 21 days (*21d*) after venous thrombosis (VT). Representative trichrome slides of WT mice at 14d after ligation (*LIG*; **B**), IL-6^−/−^ mice at 14d after LIG (**C**), WT mice at 21d after LIG (**D**), and IL-6^−/−^ mice at 21d after LIG (**E;** n = 4-6; ∗*P* = .0138).

**Fig 2:**
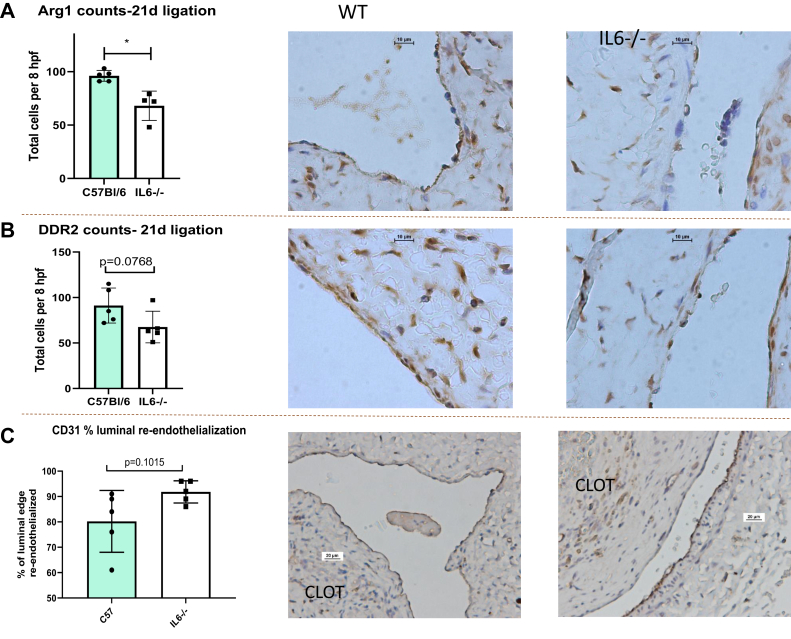
**A,** Anti–arginase-1 (*Arg1*) immunohistochemistry (*IHC*) staining results (n = 4-5; ∗*P* = .0210) and representative slides. **B,** Anti-DDR2 IHC staining results with negative antibody controls included. Original magnification ×1000 for all representative images (oil immersion). **C,** Anti-CD31 IHC staining analysis results. Original magnification ×400 (n = 4-5 for all stains).

We used the vein wall thickness as a primary measure of the post-thrombotic vein wall response or injury.^28^ In the stasis VT model, we found that the vein wall thickness (trichrome stain) was significantly less in IL-6^−/−^ mice than in the WT mice (Fig 1). The number of Arg1^+^ cells, a marker for prohealing monocytes and macrophages, was less in the IL-6^−/−^ model (Fig 2, *A*). The number of DDR2^+^ cells, a marker for fibroblasts, was less in the IL-6^−/−^ vein walls compared with the WT vein walls (Fig 2, *B*). In contrast, the number of FSP-1^+^ cells (another fibroblast marker) did not differ between the WT and IL-6^−/−^ mice (68 ± 11 cells/8 high power fields [HPF] vs 72 ± 2 cells/8 HPF; *P* = .42). We found that the number of CCR2^+^ cells, a marker for proinflammatory monocyte/macrophages, was not significantly different at 21 days (WT, 89 ± 17; vs IL-6^−/−^, 76 ± 13; n = 4-5 cells/8 HPF; *P* = .30). At 21 days, we found no significant difference in the VT area (1.67 vs 2.4 × 10^5^ mM^2^; n = 4-5; *P* = .08).

**Fig 3:**
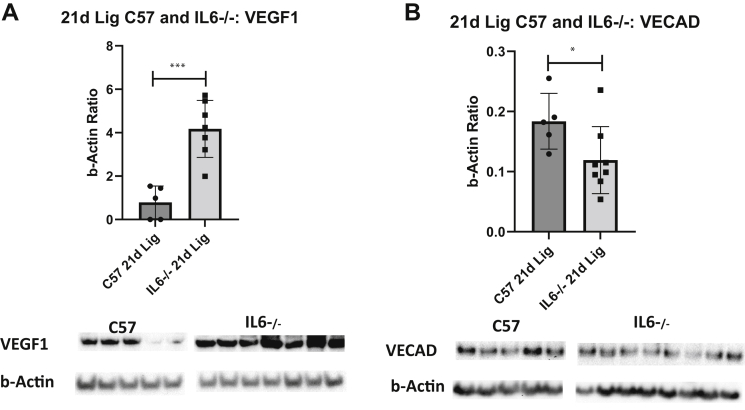
Western blot analysis. **A,** Vascular endothelial growth factor 1 (VEGF1) protein relative to β-actin in 21-day (*21d*) vein wall tissue. Blot images shown below graphs. ∗∗∗*P* = .0002. **B,** vascular endothelial cadherin (VECAD) protein relative to β-actin (∗*P* = .04; n = 5-8 for all blots).

Comparing luminal reendothelialization after thrombosis in the WT and IL-6^−/−^ mice, we found a trend toward greater luminal endothelialization in the IL-6^−/−^ mice than in the WT mice (Fig 2, *C*). Consistently, the amount of VEGF1 found in the IVC plus clot protein was higher in the IL-6^−/−^ mice than in WT mice. In contrast, the amount of VECAD protein was significantly less in the IL-6^−/−^ mice than in the WT mice (Fig 3).

### LMWH pre- and post-treatment effects in VT

**Fig 4:**
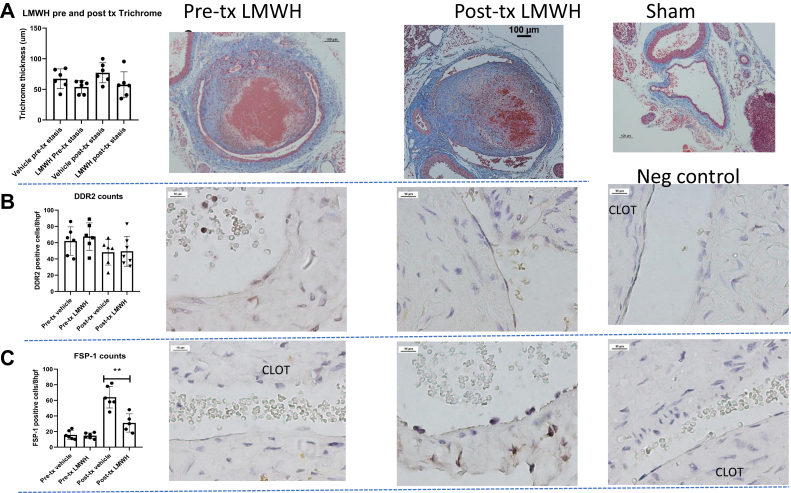
Pretreatment (*Pre-tx*) vs post-treatment (*Post-tx*) of venous thrombosis (VT) and representative images. **A,** Graph showing trichrome thickness of low-molecular-weight heparin (LMWH) administration before and after treatment (*pre and post tx*). Representative images shown to the right, with image after sham surgery shown as control. **B,** Graph showing anti-DDR2 immunohistochemistry staining results. Representative images shown to the right, with image of negative (*Neg*) control. **C,** Graph showing anti–FSP-1 immunohistochemistry staining results, with representative images shown to the right (∗*P* = .0330; n = 5-7).

No changes were found in the vein wall thickness (Fig 4, *A*) or IL-6 plasma levels at 21 days (data not shown) with either pre- or postoperative LMWH treatment. The number of DDR2^+^ cells was not significantly different comparing vehicle and LMWH, either before or after treatment (Fig 4, *B*). However, the number of FSP-1^+^ cells was significantly reduced with LMWH treatment (Fig 4, *C*).

### Short-term effects when modulating IL-6 with pretreatment medication

**Fig 5:**
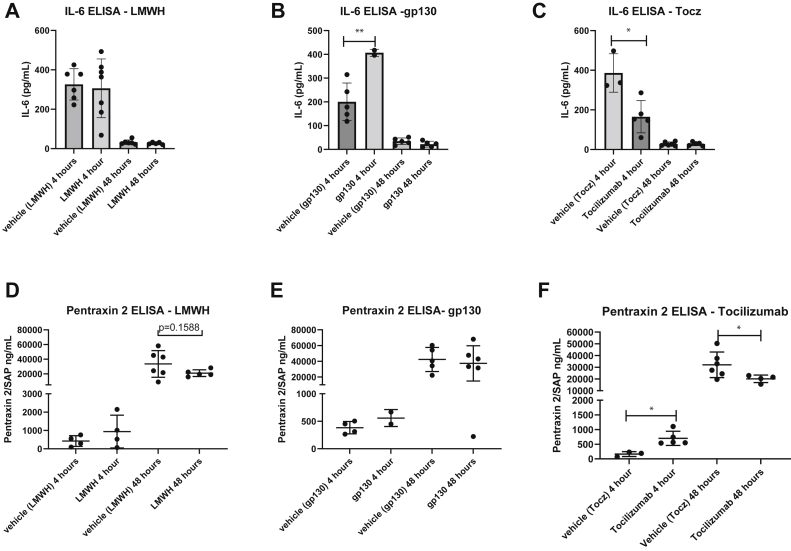
Graphs showing short-term agent circulating biomarker effects determined by enzyme-linked immunosorbent assays (ELISAs). **A,** Interleukin-6 (IL-6) level stratified by low-molecular-weight heparin (LMWH) group (n = 5-11). **B,** IL-6 level stratified by glycoprotein 130 (gp130) group (n = 3-7; ∗∗*P* = .0033). **C,** IL-6 level stratified by tocilizumab (Tocz) group (n = 3-7; ∗*P* = .0333). **D,** Pentraxin 2 level stratified by LMWH group (n = 4-6). **E,** Pentraxin 2 level stratified by gp130 group (n = 2-6). **F,** Pentraxin 2 level stratified by tocilizumab group (n = 3-7; ∗*P* = .0357, 4 hours; ∗*P* = .0438, 48 hours).

LMWH was not associated with a significant elevation in circulating IL-6 at 4 or 48 hours (Fig 5, *A*). Exogenous gp130 administration was associated with elevated circulating IL-6, consistent with the binding of the IL-6 receptor in circulation at 4 hours^37^^,^^45^^,^^46^ (Fig 5, *B*). Tocilizumab was associated with lower IL-6 plasma levels at 4 hours compared with vehicle (Fig 5, *C*). Pentraxin 2 (serum amyloid P component) is an acute phase reactant in mice and was not affected by LMWH or gp130 administration (Fig 5, *D* and *E*). Pentraxin 2 was attenuated by tocilizumab treatment at 48 hours, although an increase in pentraxin 2 was found at 4 hours (Fig 5, *F*).

### Long-term effects when modulating IL-6 with medications that inhibit IL-6 signaling after ligation treatment

**Fig 6:**
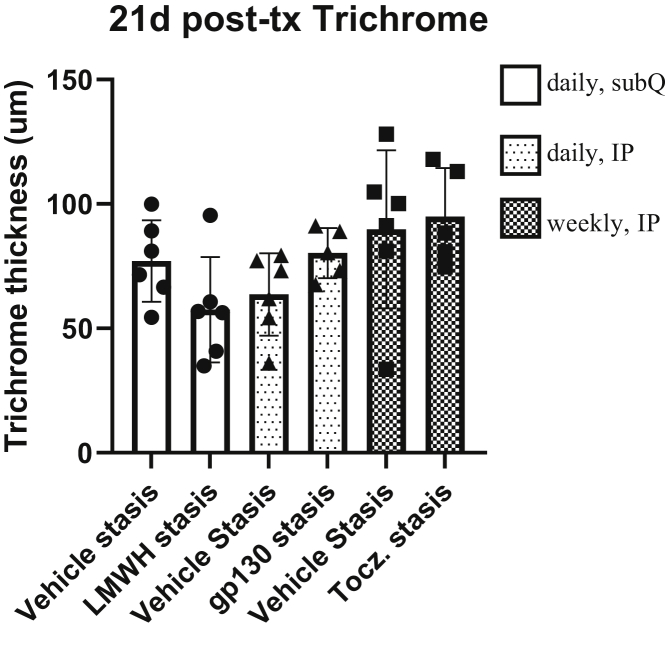
Trichrome thickness of low-molecular-weight heparin (LMWH), glycoprotein 130 (gp130), and tocilizumab (*Tocz.*) groups at 21 days (*21d*) in post-treatment (*post-tx*) groups, with no significance between groups. *IP,* Intraperitoneally; *subQ,* subcutaneously.

Given the biologic effects of the anti–IL-6 agents, gp130 and tocilizumab, we tested these agents in our stasis model of VT after a thrombus had formed to mimic human translation of post-DVT therapy. We found these agents had no significant effects on vein wall thickness or endothelial luminal coverage at 21 days (Fig 6). Furthermore, these agents had had no effects on thrombus resolution at 21 days (Supplementary Fig).

## Discussion

Because PTS has no direct prevention or cure, an important need exists to address this medical burden. Human clinical biomarker studies have suggested that elevated plasma IL-6 levels in those with DVT is associated with the development of late PTS.^9^ This stimulated our interest in whether the IL-6 signaling pathway (cis- and transcellular pathways) would confer an effect on late vein wall injury.^29^^,^^47^ In the present study, we found that the use of global IL-6 knockout mice at a chronic time point was associated with less late vein wall fibrosis, with findings consistent with those from prior studies at a slightly earlier time point.^12^ However, within the limits of this VT model, exogenous anti–IL-6 directed medications were not associated with a decrease in vein wall fibrosis.

Prior work with anti–IL-6 cytokine inhibition showed impaired vein wall influx of macrophages, less fibrosis, and accelerated remodeling.^12^ Our study also showed that *Arg1* expression was less prevalent in the vein wall of the IL-6^−/−^ group, suggesting that the monocyte phenotype might be relevant to vein wall injury and the VT model.^43^^,^^48^ The VECAD and VEGF1 results showed the presence of a coordinated differential endothelial cell response in the IL-6^−/−^ mice, supporting a remodeling effect.^49^, ^50^, ^51^ These mice are known to have impaired immune and acute phase responses, although it is a mixed immunosuppressed state of increased hypersensitivity.^52^^,^^53^ This model showed that if the cis- and transcellular signaling pathways of IL-6 have both been blocked, a decrease will occur in late vein wall fibrosis, with a trend toward increased endothelial repair. However, our techniques did not allow us to determine whether the endothelial cells were from the native vein wall endothelium or regenerated endothelium.

We have previously investigated the effects of LMWH on vein wall injury in the stasis model of VT in the mouse. In contrast to the present study, in our previous study, we found decreased vein wall thickness at 14 days and no effect on vein wall monocyte influx with LMWH pretreatment.^28^ This likely resulted from changes in how we characterized fibrotic injury to include a measurement of thickness compared with a subjective “fibrosis score.” Pretreatment with LMWH, which is the case with DVT prophylaxis, showed little differences from the post-VT treatment in terms of vein wall injury. Consistent with prior studies, we found that LMWH did not accelerate VT resolution, likely owing to the full ligation aspects of the stasis model.^28^ Nonetheless, a precedent exists for prevention of PTS in the Home-LITE trial (home therapy of VT with long-term LMWH vs usual care), in which patients had less sequelae of PTS after treatment with extended tinzaparin (similar to the sodium enoxaparin we used, another LMWH) than with short-term LMWH and warfarin counterparts after 12 weeks.^54^^,^^55^

Although the anti–IL-6 medications tocilizumab and gp130 had an early systemic effect after VT (at 4 and 48 hours), defining the ultimate dosing schedule, they did not alter the long-term vein wall fibrotic injury. Our doses and routes were determined from experimental models of rheumatoid arthritis^46^ and pulmonary fibrosis.^35^^,^^36^^,^^39^ However, a different agent, dosing schedule, or period of evaluation or more directed inhibition of transcellular pathway of IL-6 signaling might have shown greater effects. Similarly, testing these agents in a stenosis or electrolytic model of VT could yield different results.^5^ In addition, none of the mouse models can truly model venous hypertension owing to the nonbipedal nature of mice.

Others have shown that IL6^−/−^ mice have impaired wound healing, with less monocyte/macrophage infiltration, neovascularization, and contraction.^56^ IL-6^−/−^ mice had a larger VT size at a 4 and 8 days in the stasis model but not at 2 days, suggesting impaired midpoint resolution but not thrombogenesis (unpublished data). Other investigators have shown that IL-6 is important for VT resolution in the stenosis (partial ligation) VT model.^13^^,^^57^ However, to the best of our knowledge, none have evaluated currently available anti–IL-6 agents or assessed the vein wall response at late time points. Our data have also underscored the inherent limitation of global IL-6^−/−^ mice in translation to exogenous agents in mice. Moreover, IL-6 has complex signaling actions and might be important for both inflammatory and anti-inflammatory activities, as suggested by our data, and others showing IL-6 inhibition might impair thrombosis.^13^

Fibroblasts are a central contributor to pathologic fibrosis in many processes.^58^ We found significantly fewer DDR2^+^ cells in the IL-6^−/−^ mice. Consistently, with either pre- or post-treatment LMWH and no effect on fibrosis, no differences were found in the number of DDR2^+^ cells in the vein wall with this agent, despite a difference in the number of FSP-1 cells. This suggests that DDR2 cells might play a more important role in the fibrotic phenotype than do FSP-1 cells.

## Conclusions

Taken together, anti–IL-6 agents play a minor role in late stasis thrombosis vein wall injury, and, although it might be useful as a biomarker in humans,^9^ it might not be a significant direct mediator of PTS. However, selective local IL-6 transcellular signaling inhibition might be an option to only affect fibrotic inflammatory effects, with inhibition of the homeostatic IL-6 cis-signaling effects.

## Author Contributions

Conception and design: AD, CL, AO, PH

Analysis and interpretation: AD, QC, AP, AO, PH

Data collection: AD, CL, QC, AP, PH

Writing the article: AD, PH

Critical revision of the article: AD, CL, QC, AP, AO, PH

Final approval of the article: AD, CL, QC, AP, AO, PH

Statistical analysis: AD, PH

Obtained funding: PH

Overall responsibility: PH

## Figures and Tables

**Supplementary Fig:**
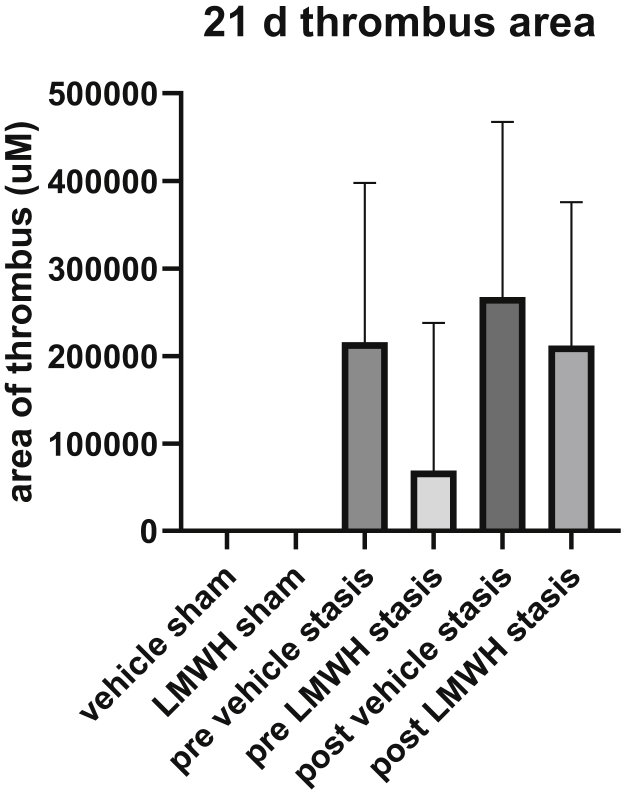
Sham, pretreatment, and post-treatment low-molecular-weight heparin (LMWH) clot area at 21 days (*d*; n = 5-7).
